# The effect of internal and external visualization of rotation on postural stability

**DOI:** 10.3389/fcogn.2024.1356441

**Published:** 2024-10-30

**Authors:** Leonardo Jost, Markus Siebertz, Philipp Hofmann, Petra Jansen

**Affiliations:** Faculty of Human Sciences, Institute for Sport Science, University of Regensburg, Regensburg, Germany

**Keywords:** spatial cognition, mental rotation, postural stability, visualization, dual-task, eye-tracking, trial design

## Abstract

**Introduction:**

During mental rotation tasks, it is assumed that participants visualize a rotation of objects in their minds (internal visualization), but mental rotation has also been linked to the visible rotation of objects on a screen (external visualization). The angular disparity in mental rotation also influences postural sway, the movements of the body center. Postural sway is thus suspected as one type of indirect measurement of the rotation process. We compare the external visualization of rotation with the suspected internal visualization during mental rotation tasks. We suspect both are similar and thus produce a comparable effect on postural sway.

**Methods:**

One hundred and fifty participants completed three rotation tasks with cube figures, two of which were aided by external visualization. Their center of pressure was measured throughout. The effects of external visualization, angular disparity, and their interaction on postural sway were compared using Bayesian statistics and a decision boundary of 3 or 1/3.

**Results and discussion:**

The results indicate no differences between conditions for all postural sway parameters. We observe differences between conditions in cognitive load and reaction time. However, as these partially also differ between the two external visualization conditions and do not transfer to differences between the postural sway parameters, the underlying processes in the three conditions are likely similar. Our results support the notion that the visualization of rotation is central to postural sway during mental rotation. This further supports that the rotation process of the external visualization and mental rotation are similar and thus that stimuli are indeed rotated mentally during mental rotation tasks. Our results further support that the common process between mental and manual rotation lies in the visualization instead of mental rotation being an imagined motor action. Because visual control and feedback play an essential role in many motor tasks, the results could also be of further interest for a more general link between motor and cognitive tasks and bidirectional benefits through the construction of visual similarities.

## 1 Introduction

The mental rotation ability describes the cognitive ability to rotate objects or images in the mind (Shepard and Metzler, 1971) and is part of the spatial abilities (Buckley et al., 2018; Linn and Petersen, 1985; Uttal et al., 2013). Spatial abilities are viewed as an essential component of intelligence and as a fundamental part of everyday life through operation and movement in the physical world (Buckley et al., 2018; Malanchini et al., 2020; Newcombe and Shipley, 2015). To reach for a cup of coffee, we need to estimate the distance and orientation of our hands to the cup. We need to remember landmarks and estimate directions to find our way in a city. Moreover, spatial abilities play an important role in visualizing data and objects through sketching or diagrams. An educational implication of scientific interest is the link of performance in these abilities with STEM (science, technology, engineering, and mathematics) performance, persistence, attainment, and achievement (Buckley et al., 2018; Shea et al., 2001; Uttal et al., 2024; Wai et al., 2009, 2010; Xie et al., 2020) in general, as well as specifics such as arithmetical reasoning (Geary et al., 2000). The link to STEM performance has also been explicitly shown for mental rotation (Hausmann, 2014; Moè, 2016; Moè et al., 2018). However, despite the relevance of mental rotation and spatial abilities, there are practical and conceptual challenges in the assessment of these abilities (Uttal et al., 2024). This is especially relevant for mental rotation as two widely used tests differ in their results regarding sex differences in performance (Jost and Jansen, 2023; Peters and Battista, 2008). The significant sex differences in one mental rotation test are also of particular interest in general cognitive research and specific STEM links (Geary et al., 2000; Halpern, 2012; Linn and Petersen, 1985; Voyer et al., 1995). However, to better link the abilities to their practical consequences, an important step is to improve the measurement of mental rotation and better understand the cognitive processes measured in the tests.

Mental rotation ability is measured through different tests, and there are some indications that the images in these tests are often rotated in the mind. However, the exact nature of this mental rotation still needs to be fully understood. We want to explore additional measurements to support or oppose the assumption that stimuli, objects, or images are rotated in the mind during mental rotation tests.

Recent studies have used measures of postural stability during mental rotation trials and have linked increased amplitudes of postural sway with larger angular disparities of mental rotation (Hofmann et al., 2023; Hofmann and Jansen, 2023). As postural stability is also affected by visualization (Lee and Lishman, 1977), we employ these measures of postural stability to compare external visualization of rotation with the suspected mental rotation process. Moreover, through this comparison, we further investigate the trial design of mental rotation tests and additionally collect eye-tracking measures.

In the following sections, we give an overview of the mechanisms by which images are suspected to be rotated in the mind, and the concurrent measures of postural sway. Before this, however, it is important to introduce some aspects of mental rotation tests.

### 1.1 Mental rotation tests and measurements

Mental rotation ability has widely been assessed through different types of tests. The traditional tests of relevance here are chronometric mental rotation tests based on the study of Shepard and Metzler (1971). They employ trials in which two figures are presented, either the same (rotated) or different (mirrored). However, Jost and Jansen (2023) demonstrate varying performance depending on the trial design. They conclude that different abilities are involved in solving mental rotation trials and emphasize that further investigation of trial designs is necessary.

In recent work on the trial design of mental rotation tests, Jost and Jansen (2020) investigated the comparison of one target with two alternatives. The two alternatives are mirrored so each target can be rotated into congruence with one alternative. This allows the inclusion of all trials in the analysis instead of distinguishing between rotated and mirrored trials, where mirrored trials are mostly discarded from analyses. Jost and Jansen (2020) also demonstrated that this type of trial produces similar behavioral results for all trials as the rotated trials of traditional tests with two figures.

It is often hypothesized that there are large sex differences in mental rotation performance (Linn and Petersen, 1985; Voyer et al., 1995). These are, however, only observed in psychometric tests. The chronometric tests, on average, produce no or much smaller sex differences (Jansen-Osmann and Heil, 2007; Jost and Jansen, 2023; Peters and Battista, 2008; Voyer et al., 1995). Thus, the primary reason for large sex differences must lie in the differences between the psychometric and the chronometric test and not in mental rotation ability, which is assumed to be involved in both tests (Jost and Jansen, 2023; Peters and Battista, 2008). Therefore, it does not seem fruitful to search for sex differences and their reasons in chronometric tests to explain the large sex differences in psychometric tests (Peters and Battista, 2008). Moreover, unless sex differences are established for one test, we must not speculate about reasons even if they occur in one experiment because they cannot be generalized to the test overall. However, as there is still only a little research investigating differences between tests, it seems helpful to document sex (non-)differences in all types of mental rotation tests.

### 1.2 Mental rotation, visual rotation, and visualization

In all mental rotation tests, it is assumed that the images, objects, or stimuli are rotated in the mind to solve the trials. We will call this rotation in the mind internal visualization. Shepard and Metzler first assumed this (Shepard and Metzler, 1971) because reaction time increased linearly with the angular disparity of figures, indicating similarities to physical rotations with a fixed rotation speed. This effect has since been widely reproduced, not always as linear, but always as monotonic, which could at least in part be due to speed-accuracy trade-offs. However, to our knowledge, the distinction between linearity and monotonicity and possible speed-accuracy trade-offs has never been further investigated.

In partial support of the assumption of internal visualization, the rotation of objects has been identified as one strategy through word protocols. Although it was the most used strategy, it was not used in all trials or by all participants (Hegarty, 2018).

Next to mental rotation trials, some studies have utilized visual rotation trials. In these trials, visual depictions of rotating objects are provided on a screen that can be objectively observed. In contrast to the internal visualization during mental rotation trials, we call this external visualization as the visual representation of the rotation is provided externally. The visual rotation can be provided in different ways. The first experiments employed manual rotation, that is, visual rotation caused manually by movements. To further increase similarities to physically rotating objects in ones hands, Wohlschläger and Wohlschläger (1998) employed rotational hand movements in manual rotation. They observed similar reaction time patterns in their manual rotation trials compared with mental rotation trials when stimuli were reoriented along the canonical axes. This was seen as evidence for similarities between mental and manual rotation.

Next to the similarities in reaction time patterns, manual rotation training has also been shown to improve mental rotation performance (Adams et al., 2014; Wiedenbauer et al., 2007). Adams et al. (2014) also showed that the training effect on mental rotation performance was not significantly different for manual and mental rotation training. Jost and Jansen (2022) investigated three different causes of visual rotation by further breaking down the manual rotations. The training effect was the same, whether the visual rotation was caused by rotational or non-rotational hand movements or simply provided automatically. It is thus suspected that the visualization of rotation is the key similarity between mental and manual rotation and not the motor action. However, there has not been any investigation of manual rotation training where the rotational hand movement did not create visible feedback.

### 1.3 Strategies in mental rotation and eye-tracking

Although rotation in the mind was identified as not the only strategy in mental rotation tests, analyses of strategies have mostly focused on this strategy. Rotation strategies can generally be separated into piecemeal and holistic rotation strategies (Khooshabeh and Hegarty, 2010). However, the identification of these strategies is only based on researchers' assumptions about how eyes should move during such strategies. No studies have linked eye movements to other forms of strategy identification, such as word protocols, and thus, there is no clear evidence-supported link between gaze patterns and strategies.

Research questions concerning gaze in mental rotation trials are mostly concerned with two topics. The first is the time course of different cognitive stages during the task. Just and Carpenter (1976, 1985) proposed linear progress through three pre-defined cognitive stages (search, transformation and comparison, and confirmation). Xue et al. (2017) also identified three stages through statistical modeling but found that the transition between stages was not linear. However, neither of these lines of work accounted for the possibility of varying rotation strategies, which by themselves would present their own gaze patterns.

The second topic of gaze analyses in mental rotation was mostly concerned with a discrimination between piecemeal and holistic rotation strategies (Khooshabeh and Hegarty, 2010; Nazareth et al., 2018; Voyer et al., 2020). However, this line of work analyzed or manipulated the gaze patterns throughout the whole trials and from this inferred rotation strategies. It is neglected that there are different cognitive stages, which are also represented by different gaze patterns.

The connection of gaze patterns with performance also ignores that gaze parameters may already correlate with performance parameters. Thus, identifying better-performing strategies through gaze parameters may be problematic. For example, the results of Nazareth et al. (2018) can be explained by speed-accuracy trade-offs instead of strategic differences. This contamination of behavioral data and strategy identification through speed-accuracy trade-offs was already identified by Liesefeld et al. (2015). Other problems arise because strategies are sometimes assumed to be one reason for large sex differences in mental rotation tests. These large sex differences occur in psychometric tests, but the gaze patterns are primarly analyzed in chronometric tests (for technological reasons). Peters and Battista (2008) already noted this problem regarding fMRI studies.

There are further possible investigations using eye-tracking. For example, Xue et al. (2017) also investigated whether the left or right figure was rotated in the mind. Paschke et al. (2012) investigated differences between rotated and mirrored trials. Similar questions could be relevant for the three-figure trial design of Jost and Jansen (2020). There, it is unclear if and to what extent both alternative figures are employed in the solution of the task. Theoretically, using only one alternative figure is sufficient to solve the task similar to either rotated or mirrored trials of the Shepard and Metzler (1971) mental rotation test. Jost and Jansen (2020) already hypothesized that participants could look at the figures from left to right according to the reading direction of their mother tongue.

In all analyses of gaze patterns, the technological limitations of eye-trackers must also be kept in mind, regarding both the spatial and temporal precision.

### 1.4 Mental rotation and postural sway

In recent studies postural sway has emerged as another possible indirect measurement of the rotation process during mental rotation trials. Postural sway describes movements of the body to maintain a standing upright posture. The underlying ability is postural stability, also known as balance, the ability to control the body's center of gravity over the supporting surface (Shumway-cook et al., 2007). For its measurement, the center of pressure (CoP) is typically calculated using a force plate (Rhea et al., 2014). There are numerous parameters for measuring postural sway, with a common classification distinguishing between global and structural parameters (Baratto et al., 2002). Global parameters assess the overall extent of the COP-trajectory, with smaller deviations typically interpreted as indicating greater postural stability (Palmieri et al., 2002; Rhea et al., 2014). Whereas, structural parameters focus on the temporal organization within the COP signal. No consensus exists regarding the use of specific parameters for particular research objectives, as highlighted by Duarte and Watanabe (2023). But to avoid complications related to multiple testing, studies must focus on a limited set of relevant parameters. Recommendations for such selections have been provided by, for example, Prieto et al. (1996), Baratto et al. (2002), and Yamamoto et al. (2015).

Regarding connections to mental rotation, studies indicate that postural stability positively correlates with mental rotation performance in children (Jansen and Heil, 2010) and older adults (Jansen and Kaltner, 2014). Furthermore, there is a neuronal overlap, as the cerebellum, which is crucial for balance, is also active during mental rotation tasks (Podzebenko et al., 2002). Several dual-task paradigms demonstrate stabilization of posture while solving mental rotation tasks (Budde et al., 2021; Burcal et al., 2014; Dault et al., 2001).

In two recent dual-task studies, Hofmann et al. (2023) and Hofmann and Jansen (2023) measured the CoP of standing participants during mental rotation trials. Mean amplitude and maximum range in the anterior-posterior and mediolateral direction of the CoP course showed a monotonic and sometimes linear increase with angular disparity in the same way as reaction time. The sway velocity was independent of angular disparity in both studies. It was assumed that the linearly increasing parameters are measurements of the rotation process, similar to reaction time. In contrast, the sway velocity cannot be regarded as a measurement of the rotation stage or aspects of it.

Because reaction time and accuracy are not easily comparable between manual, mental, and visual conditions due to the varying visual aids, these postural sway measurements could offer additional possibilities to compare conditions. There is also a possible link to the visualization because information from the visual system plays an essential role in postural control, and even watching the scene of a rotating abstract image influences postural sway (Lee and Lishman, 1977; Redfern et al., 2001; van Asten et al., 1988). In this way, the effect of external visualization of rotation on postural sway is demonstrated. It is suspected that the internal visualization of rotation during mental rotation trials causes the same effect. This is supported by the observation that larger angular disparity produces a larger postural sway.

Difficulties regarding the comparison of postural stability between conditions, however, arise as it is affected by the difficulty of the concurrent cognitive task (Pellecchia, 2003). Moreover, reaction time also affects mental rotation (Hofmann et al., 2023). It is thus necessary to control effort and reaction time to isolate other effects on postural stability.

### 1.5 Goal of the study

The current study's goals are 2-fold. The main goal is to establish similarities and differences between mental and visual rotation conditions through measures of postural sway. The secondary goal is to investigate aspects of the trial design.

As the visualization of rotation seems to be an essential mental rotation process, we want to compare the external visualization of rotation on a screen with the internal visualization of rotation during mental rotation tasks. If there is indeed a similarity between external and internal visualization, it is suspected that both produce a comparable effect on postural stability. This could further serve as an explanation for the observed effect of mental rotation on postural sway.

We aimed to reduce the effect of differing cognitive effort and reaction time between conditions on the interpretation of results by implementing two visual rotation conditions intended to differ in cognitive effort and reaction times. In one condition, similar to the training condition used by Jost and Jansen (2022), the target figure rotates until it reaches congruency with one alternative. Because the rotation stops after congruency is reached, there is theoretically no necessity to observe the rotation or the stimuli before that. To counteract this possibility, we employed a second visual rotation condition. In this condition, the target continuously rotates and does not stop. Because of the increased complexity of comparing the figures, we also assumed increased cognitive effort and reaction times in this condition. This could help to exclude differing effort and reaction times as sole reasons for observed differences between the first visual rotation condition and the mental rotation condition. We also measure physical effort using subjective measures and cognitive effort using both subjective and objective measures.

A long-used objective measure of cognitive load is pupil size during cognitive tasks (Beatty and Lucero-Wagoner, 2000; Kahneman et al., 1969), which has already been used during mental rotation trials (Bauer et al., 2021, 2022; Bochynska et al., 2021; Campbell et al., 2018). However, whether pupil diameters increase with angular disparity is unclear from those studies. In contrast to the other studies, Bochynska et al. (2021) report no increase with angular disparity. But their trials were always shown for a fixed duration of 4,000 ms. They subsequently did not observe the monotonic increase of reaction times, possibly due to speed-accuracy trade-offs, as their median accuracy reached even below 0.6 for 180° of rotation (This might generally be problematic for interpretation, see Jost, 2021). This underlines the importance of test protocols. Differences could also arise due to different preprocessing and baseline corrections, which greatly influence outcomes (Mathôt et al., 2018). The concurrent measurement of gaze data allows us to investigate the trial design readily. In our case, the investigation of gaze on the target figure was also planned as a manipulation check as it is the only visual difference between conditions.

### 1.6 Hypotheses

Because the study's goal and our predictions concern not only differences between conditions but also similarities, we will use Bayesian statistics. In contrast to null-hypotheses significance testing, this allows evidence to be gathered for the null hypothesis. Nevertheless, the hypotheses are formulated as alternative hypotheses predicting differences. The following hypotheses were preregistered.

Main hypothesis: There is a difference in postural sway during mental and visual rotation tasks. This is further quantified by an interaction with angular disparity.

Because postural sway measurements are affected by cognitive effort, physical effort, and reaction time, we investigated secondary hypotheses regarding differences between conditions in effort and reaction time to support the interpretation of results regarding the main hypothesis. The measurement of pupil diameter also offers the possibility of investigating the effect of angular disparity on cognitive effort. The visual rotation with stop is expected to require less cognitive effort because only two similar figures must be compared, similar to a 0°-trial in mental rotation. Because the difficulty after the stop is independent of the starting angle, no additional mental effort may be required for larger angular disparities, and we thus expect a smaller effect of angular disparity on cognitive load in this condition. The visual rotation without stop was designed to increase cognitive effort to a comparable level to mental rotation.

For reaction time, we aim to replicate the well-known effect of angular disparity (Shepard and Metzler, 1971). This is also expected for the visual rotation conditions similar to the effect found for manual rotation (Wohlschläger and Wohlschläger, 1998). However, as the rotation speed is fixed in the visual rotation conditions, the angular disparity effect may be similar to the fixed rotation speed and different from the mental rotation speed (for differences between visual and manual rotation, see Jost and Jansen, 2022).

The following secondary hypotheses were investigated:

S1: There is a difference in cognitive effort between the mental and visual rotation conditions (subjectively and objectively).

S2: There is a difference in physical effort between the mental and visual rotation conditions (subjectively).

S3: There is a monotonic increase in reaction time with increasing angular disparity. This effect differs between the mental and visual rotation conditions.

S4: Pupil diameter increases with angular disparity in all trials. This increase is larger for the mental rotation compared with visual rotation.

The gaze data also offers possibilities for further investigation of the mental and visual rotation trial design. We thus planned to investigate whether and in which order participants view the three figures. Because we did not preplan statistical analyses for this, we preregistered the hypothesis as exploratory:

E1: Participants look at all three figures during the mental and visual rotation tasks. We will also examine the time course/order in which the figures are viewed (viewing the left alternative before the right, focusing more on the correct alternative at the end of the trial).

In support of the documentation of varying sex differences in varying mental rotation tests, we also describe the sex differences in the behavioral data of our experiment. In chronometric tests, there are on average no meaningful sex differences in performance. We therefore did not expect sex differences here and there was no theoretical hypothesis associated with this analysis. This descriptive analysis was not preregistered.

## 2 Materials and methods

### 2.1 Participants

Overall, 150 persons (77 men, 72 women, one other; mean age 22.7 ± 2.6 years; 85 with previous experience with mental rotation) participated in the study. Our sample included participants with previous experience with mental rotation because it is assumed that the underlying mechanisms should not change with experience. The sample size was determined by Bayesian statistics. Data collection was planned to stop if the Bayes factors for all hypotheses reached a decision boundary of 3 or 1/3 or if at most 150 participants were tested. The maximum sample size of 150 participants was chosen for feasibility reasons but should exceed recommendations for sufficient power for frequentist analyses of within-subject designs (Brysbaert, 2019; Brysbaert and Stevens, 2018). Bayes factors were initially checked after 30 participants were tested and again in steps of 10 participants. At least one Bayes factor of interest remained inconclusive for all analyses.

Participants were recruited by advertisement in the newsletter for students (Bachelor Angewandte Bewegungswissenschaften) at the University of Regensburg. Participants received study credit for participation. Participants had to be at least 18 years old. Exclusion criteria were diseases or injuries affecting the balance. The study was approved by the Ethical Board of the University Clinic of Regensburg.

### 2.2 Postural sway

Prieto et al. (1996) identified groups of highly correlated parameters (*r* ≥ 0.9) of postural sway. To prevent ourselves from redundant parameters, we selected each of our four parameters from distinct groups. While selecting only a few parameters will not fully capture the complexity of “Postural Stability,” we believe the chosen parameters are suitable for this study, as this selection builds on the findings from two prior experiments investigating postural sway during mental rotation trials (Hofmann and Jansen, 2023; Hofmann et al., 2023), ensuring optimal comparability across the studies.

**Figure 1:**
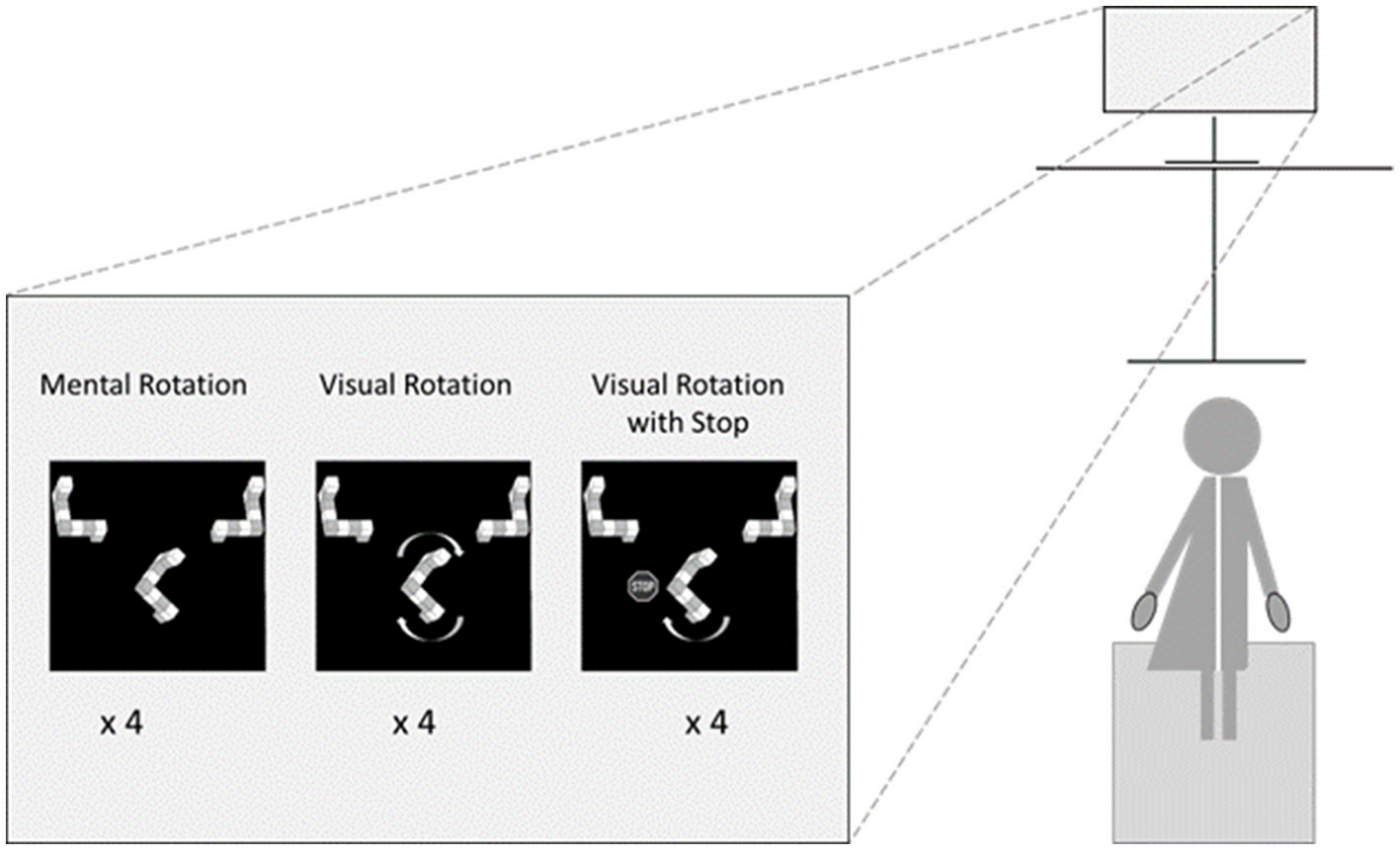
Experimental set-up.

Participants stood ~70 cm in front of the screen on a force plate (AMTI OR6-2000, 1,000 Hz). The software Vicon Nexus 2.13 controlled the force plate, and the center of pressure was continuously recorded (see Figure 1). Four global parameters were calculated for each correctly answered mental or visual rotation trial by the center of pressure course over time: the mean amplitude [mm] (Hufschmidt et al., 1980), the maximum range in anterior-posterior direction [mm], the maximum range in mediolateral direction [mm], and the mean sway velocity [mm/s]. Higher values in these measurements are interpreted as less postural stability (Palmieri et al., 2002).

### 2.3 Mental and visual rotation

For all mental and visual rotation tasks, we used the three-figure layout of Jost and Jansen (2020) with two alternatives on the top left and right and the target in the center of a Philips 272S (27”, 1920x1080) screen. Horizontal distances to the left and right from the center were 500 px; vertical distances were 200 px. Participants had a Bluetooth mouse in each hand and should press with the hand's index finger corresponding to the congruent alternative. We used three types of trials: mental rotation, visual rotation (without stop), and visual rotation with stop.

In the mental rotation trials, participants had to rotate the target mentally.

In the visual rotation trials, targets remained fixed for 500 ms and then turned by 3°/frame, starting in the shorter direction, to reach congruence with one alternative as quickly as possible. For angular disparities of 180°, the direction was randomly chosen. The target kept turning until the trial was completed. At the frame rate of the implemented experiment, this resulted in rotation speeds of 35–40°/s.

The visual rotation with stop trials was similar to the visual rotation trials. The only difference was that the visual rotation stopped once congruence with one alternative was achieved.

In all trials, participants had to decide whether the target matched the left or right alternative by pressing the mouse in the respective hand with their index finger. Trials were presented until an answer was given. Reaction time and accuracy were measured for each trial.

Stimuli were taken from the library of Jost and Jansen (2020) using the parameters described in Table 1. Models 2–8 and 12–16 by Peters and Battista (2008) were used because models 1, 9, 10, and 11 can be transformed into other models by mirroring and/or rotating. Figures were rotated in steps of 45° around the y-axis for the starting angles and in steps of 3° for the presentation of visual rotation. No figures with a starting angle of 0° were used.

**Table 1:** Parameters for generation of stimuli.

**Parameter group**	**Parameter**	**Value**
Color options	Background color	Transparent (black)
	Border color	Black
	Face color	Gray, white
Sizing and formatting	Cube diameter	70 px
	Image size	600 px^*^600 px
	File format	png
	Centering	Optical
Model properties	Base orientations	a,b
	Models	Peters and Battista (2008), 2–8, 12–16
	Base rotation angles (x, y, z)	−15°, 0°, 15°
	Angle difference	45°
	Rotation axes	y

Targets were presented in random order such that each choice of parameters occurred again only after all other choices had occurred at least once. As angles are merged by the direction/shortest distance for analysis (i.e., 45° and 315° are both a rotation of 45°), rotations in different directions were treated as the same parameters such that all angles (45°, 90°, 135°, and 180°) occurred equally often. Between reoccurrences of the exact same target, at least 10 different targets were shown (in this case, different directions were treated as different targets except for 180°). Independent of the targets, the orientation of the alternatives was randomly chosen for each trial. The randomization of trials and stimulus order was performed using shuffling in Python.

After all trials, there was a 1,000 ms break. In the practice blocks, participants got feedback during this break (✓—correct, *X*—wrong). During the experimental blocks, a fixation cross (+) was shown.

### 2.4 Eye tracking

Gaze and pupillary data were collected using the PyGaze package (Dalmaijer et al., 2014) and an SMI RED250mobile eye tracker mounted below the screen.

Pupil diameter [mm]^1^ was measured for each trial frame to measure objective cognitive effort. Frames with smaller pupil diameters than 1 were excluded from all eye measurements. Blinks yield a pupil diameter value of 0 whereas typical measurements are >3, small values are excluded as possible indicators of beginning or ending blinks or otherwise invalid measurements (Mathôt et al., 2018).

To calculate the average pupil size of each trial, the sum of pupil diameters of each frame was divided by the total number of frames within the trial. The baseline at the start of each trial (median of the first 3 frames, equivalent to around 200–250 ms) was used for subtractive baseline correction (Mathôt et al., 2018).

Furthermore, gaze data was recorded for all cube figures of each trial. For each frame, gaze focus was counted on one figure if the focus was within 320 px to the center of the respective figure such that no pixel can be counted as focus on two figures (the distance between the centers of the nearest figures is >640 px). For each figure, the sum of frames with a focus on the respective figure was counted.

The gaze and pupil diameter measurements are limited because they are restricted in frequency to the visual rotation display. Testing yielded a frame rate of around 13 Hz (ca. 70–80 ms per frame). The average pupil diameter should not be majorly affected, and the gaze data was analyzed exploratively, which should be sufficient to determine whether all figures are looked at. This limitation thus seemed acceptable as these measurements are not relevant to the main hypothesis while still providing some insights.

### 2.5 Subjective effort

Ratings of perceived exertion (RPE; both physical and cognitive) were measured via the Anstrengungsskala Sport (Büsch et al., 2022), ranging from 1 to 10. We included the color scheme and the additional option “0” for resting states included by (but not assigned with the value 0) (Büsch et al., 2015). Participants were presented with the RPE scale and answered verbally. Cognitive RPE was recorded before physical RPE.

### 2.6 Demographic data

Demographic data was collected using a digital questionnaire. Participants indicated their sex (male, female, diverse), age (in years), and previous experience with mental rotation (yes or no).

### 2.7 Study design and procedure

The study employed a within-subjects design with three conditions (mental rotation, visual rotation, and visual rotation with stop). Participants were tested individually. They were informed about the study procedures and then completed three practice blocks (one of each condition) and 12 main blocks (four of each condition) while standing on a force plate. Before each block, participants were instructed about the condition. Practice blocks took 30 s each with a self-selected break (at least 10 s) between blocks, and the main blocks took 70 s each with a break of 90 s. Blocks were not interrupted during trials when the time was up and lasted until the last trial was answered. Afterward, demographic data was collected. The practice blocks and the 12 main blocks were presented in random order using randomization in OpenSesame.

Stimulus presentation, response handling, and logging were controlled with the OpenSesame software (version 3.2.8 using python 2.7 in 32 bit to accomodate the eye tracker, Mathôt et al., 2012). The program was presented on a 27” screen on a platform adjusted to each participant's height. Participants stood ~70 cm in front of the screen on a force plate in a two-legged close stance. To standardize the foot position, participants were positioned on the force plate along a taped “T” of 3 cm wide tape on the force plate. Participants wore ultra-thin socks to ensure better hygiene while mimicking a barefoot condition. Participants were instructed to stand as still and upright as possible and not to speak during the tasks. The arms should hang relaxed by their sides, and the palms of their hands should face the body.

Because the position of the eye tracker, monitor, and the participants was standardized during the whole experiment, the eye tracker was calibrated only at the beginning of the experiment. The force plate was recalibrated before each block to avoid drifting effects.

### 2.8 Outliers and data exclusion

In line with the work of Hofmann and colleagues (Hofmann et al., 2023; Hofmann and Jansen, 2023), all incorrectly solved trials, and the first and last 5 s of each block were excluded from further analysis of the postural data because of possible movements due to anticipating the start or end of a block. We failed to state this in the pre-registration. Due to technical problems, the force plate data of seven participants were excluded. If there were irregular movements during the measurement (speaking, coughing, scratching, etc.), this was noted, and the respective block was excluded from analyses of postural sway. Also, 442 mental rotation trials were excluded because participants answered before the trials were shown by keeping mouse buttons pressed throughout the breaks.

We then detected and excluded outliers in line with standard procedures. Mental and visual rotation trials with reaction times more than 3 standard deviations above or below the respective mean for the angular disparity in each condition were excluded (502 out of 39,750 mental rotation trials, 366 out of 25,853 force plate measurements). Two participants who performed at or below the chance level in any cognitive task were excluded from all analyses.^2^ Wrongly answered trials were excluded from reaction time and pupil diameter analyses.^3^

We preregistered to treat angular disparity as a discrete variable, with the necessity of imputing missing values for any combination of angular disparity and condition. As we were able to treat angular disparity as a numerical variable, we did not need to impute it anymore. Moreover, we planned to exclude participants with missing data for two or more rotation angles in any one condition. We planned additional analyses if 10% or more of the participants needed imputation. Only one participant had missing data for one angle in one condition, and no participants had missing data for more than one angle.

Trials in which no gaze points on the stimulus figure or neither alternative are recorded were planned to be inspected for possible exclusion. The inspection, however, revealed that accuracy in these trials was relatively high (91.4% for trials without gaze points on the target and 85.8% for trials without gaze points on the alternatives compared with 91.8% for all trials). This suggests that both the alternatives and targets were observed, just not measured by the eye tracker. This could be due to general technical limitations of the eye tracker and specific to our low resolution of recordings. Moreover, unrealistically small baseline pupil sizes based on a visual inspection of a histogram were planned to be excluded from pupil size analyses (Mathôt et al., 2018). This did not occur, probably because small pupil size measurements were already excluded. The histogram is included in the Supplementary material.

### 2.9 Statistical analysis

Analysis was performed using Bayes factors for linear models. Analysis was carried out in R (version 4.3.1, R Core Team, 2023) using the BayesFactor package (version 0.9.12-4.4, Morey and Rouder, 2022). For the main hypothesis, the four postural parameters, mean amplitude, sway velocity, and both sway ranges, were dependent variables in separate linear models. For Hypothesis S1, cognitive effort rating and pupil size served as dependent variables; for Hypothesis S2, the physical effort rating; for Hypothesis S3, the reaction time of mental rotation trials; and for S4, the pupil size was again investigated.

For all analyses, we compared models with the main effect of angular disparity or condition, the model with both main effects, the model with the additional interaction effect, and the model using only the intercept between participants. The effects of angular disparity were not compared for subjective cognitive and physical effort because they were not computed on a by-trial basis. The angular disparity was treated as a numerical variable and condition as a factor with three levels (mental rotation, visual rotation, and visual rotation with stop). Bayes factors above 3 and below 1/3 were viewed as conclusive evidence. Bayes factors between 3 and 1/3 were viewed as inconclusive, supporting neither the null nor the alternative hypotheses. For reasons of symmetry and due to large values, we report the decimal logarithm of the Bayes factors. The decision boundaries are thus converted to 0.477 and −0.477, respectively. We used two-tailed tests. We did not correct for multiple comparisons.

The evidence for or against the hypothesis-relevant main effects was quantified by comparing the best-fitting model containing this main effect but no higher-order interaction with the models, including or excluding the effect in question. For the interaction, we compared the model with both main effects with the model that additionally contains their interaction.

We report the model most strongly favored by the Bayes factors for each dependent variable. If Bayes factors favored a model including the factor condition or the interaction, follow-up analyses were conducted to see how the three conditions differ. For each pairwise comparison between two conditions, we used the linear models described above, leaving out the data for the third condition. We used the fixed-effects structure of the best-fitting model and compared it with a model excluding the condition or the interaction.

The BayesFactor-package places a Cauchy distribution on the effect size to calculate Bayes factors. Its scaling factor r was set to the default values of 0.5 for fixed effects (Angle of Rotation and Condition) and 1 for random effects (variance between participants). For fixed effects—which are relevant to our hypotheses—this means that the prior for the alternative hypothesis (that there is a difference between conditions in the DV) models the range of expected effect sizes in a way that the expected effect size lies with a probability of ~80% between −1.5 and 1.5 (Schmalz et al., 2023). According to van Ravenzwaaij and Wagenmakers (2022) default priors offer certain advantages that outweigh their drawbacks. Additionally, we calculated robustness regions. As the width parameter of the Cauchy distribution needs to be >0 and is technically not limited upwards, we checked the range of 0.01 (just above 0) to 1 for changes in the qualitative conclusion if a different prior scaling factor had been chosen. A scaling factor of 1 models the range of expected effect sizes so that the expected effect size lies with a probability of ~80% between −3 and 3 (Schmalz et al., 2023).

We originally preregistered to use ANOVA for the overall comparison and *t*-tests for pairwise comparisons. We switched to linear models because they allowed us to account for the numerical properties of angular disparities and to account for the complete data structure instead of comparing averages. This change was made during data collection but before the full sample analysis. Nevertheless, we still calculated the preregistered analysis. We report in the results section whenever the analyses differ with regard to the direction of evidence and report the full results of the preregistered analysis in the Supplementary material.

## 3 Results

**Figure 2:**
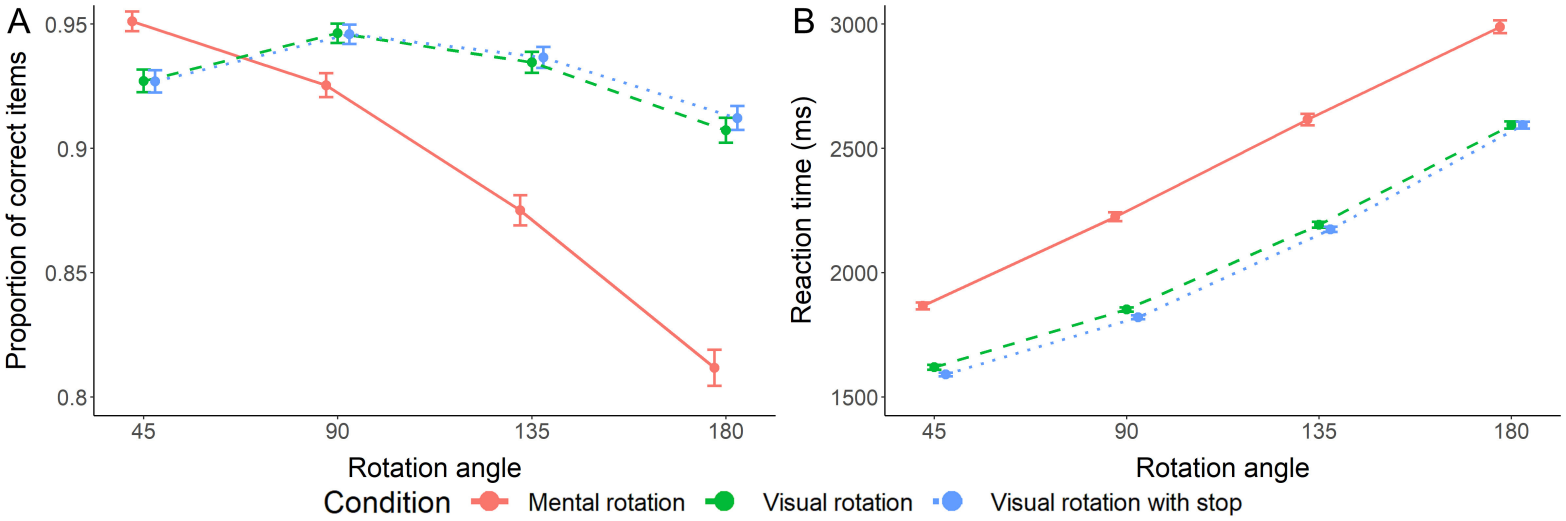
Accuracy **(A**, Left**)** and reaction time **(B**, Right**)** of rotation trials. Error bars represent the standard error.

Before starting with the planned analyses, it is essential to note that average accuracy in all conditions was sufficiently high and in line with the results of other mental and visual rotation studies. Accuracy was lowest for 180° angular disparity in the mental rotation condition but exceeded 0.8 (see Figure 2A).

### 3.1 Main hypothesis: parameters of postural sway

#### 3.1.1 Mean amplitude

**Figure 3:**
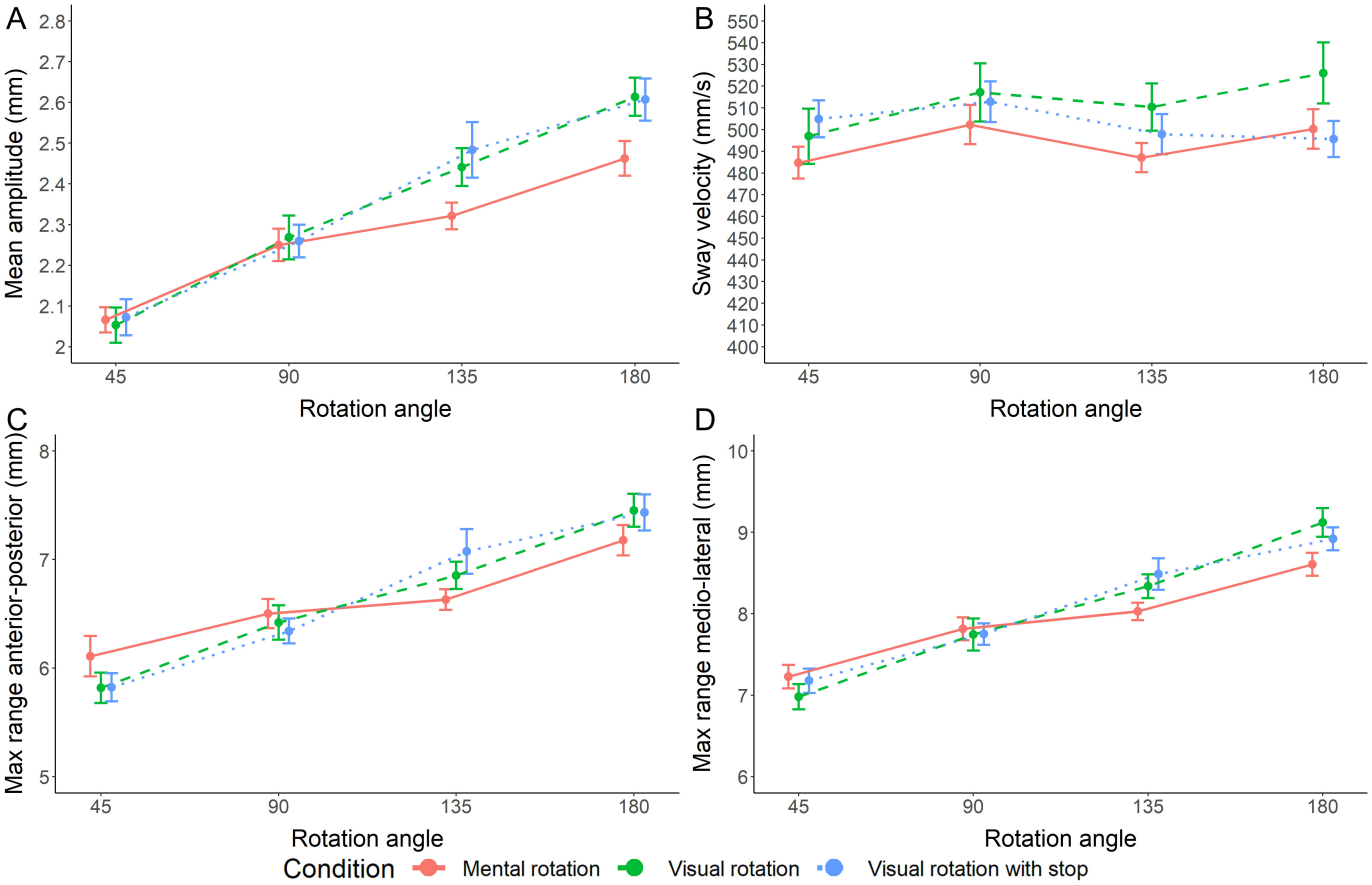
**(A–D)** Measures of postural stability during rotation trials. Error bars represent the standard error.

Analysis of mean amplitude preferred the model containing only the main effect of angular disparity [all log_10_(BFs) ≥ 3.301, RR = [0.02, >1]]. The model containing both main effects was also preferred over the interaction [log_10_(BF) = 2.558, RR = [ < 0.01, >1]]. This implies decisive evidence against a condition difference as a main effect and an interaction with angular disparity (see Figure 3A).

#### 3.1.2 Sway velocity

Bayes factors strongly suggest that sway velocity does not depend on angular disparity or condition [all log_10_(BFs) ≤ −1.410, RR = [0.17, >1]]. The model containing both main effects was also preferred decisively over the interaction [log_10_(BF) = 3.170, RR = [ < 0.01, >1]; see Figure 3B].

#### 3.1.3 Maximum range in anterior-posterior and medio-lateral direction

Like mean amplitude, the maximum range in anterior-posterior direction is decisively implied to depend only on angular disparity but not on condition [all log_10_(BFs) ≥ 2.279, RR = [0.05, >1]] as is the maximum range in medio-lateral direction [all log_10_(BFs) ≥ 3.240, RR = [0.02, >1]]. In both cases, the model containing both main effects was also preferred decisively over the interaction [log_10_(BF) = 2.161 and 2.281, RRs = [< 0.01, >1]; see Figures 3C, D].

### 3.2 Secondary hypotheses

#### 3.2.1 Hypothesis S1: subjective cognitive effort

**Figure 4:**
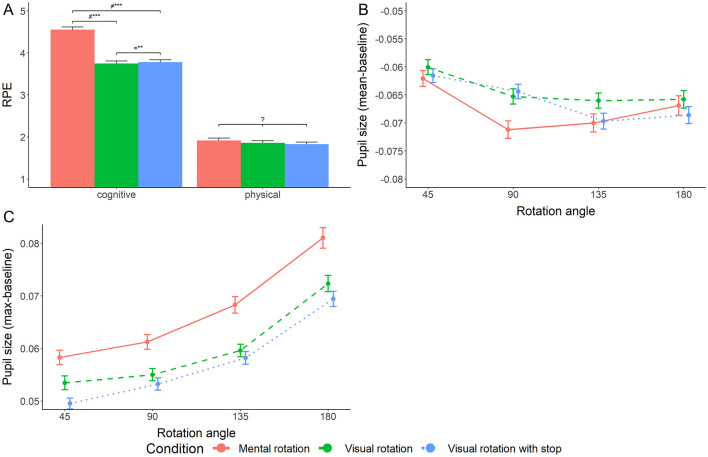
Measures of effort: subjective cognitive and physical effort after rotation blocks **(A**, Left**)** and average **(B**, Center**)** and maximal **(C**, Right**)** pupil size changes during rotation trials. Error bars represent the standard error. Stars mark the magnitude of the Bayes factor suggesting differences (≠), no differences (=) or inconclusive results (?). *BF > 3, **BF > 10, ***BF > 100.

Decisive evidence for cognitive effort to differ between conditions results from our analysis [log_10_(BF) = 86.196, RR = [< 0.01, >1]]. Pair-wise comparisons indicate decisive evidence for differences between the mental rotation condition and both the visual condition [log_10_(BF) = 63.196, RR = [< 0.01, >1]] and the visual-with-stop condition [log_10_(BF) = 60.369, RR = [< 0.01, >1]] but strong evidence against a difference between the two visual rotation conditions [log_10_(BF) = −1.048, RR = [0.12, >1]; see Figure 4A].

#### 3.2.2 Hypothesis S1/S4: pupil diameter/objective cognitive effort

The substantially preferred model for baseline-corrected mean pupil size includes both main effects of angular disparity and condition but no interaction [all log_10_(BFs) ≥ 0.941, RR = [< 0.01, 0.85]]. Pairwise comparisons revealed decisive evidence for a difference between the mental and visual rotation conditions [log_10_(BF) = 2.286, RR = [< 0.01,>1]]. Evidence regarding the difference between the mental rotation and the visual rotation with stop remains inconclusive [log_10_(BF) = −0.389, RR = [0.06,0.61]], while substantial evidence indicates no difference between the two visual rotation conditions [log_10_(BF) = −0.842, RR = [0.22,>1]]. The pairwise differences between conditions differed in the preregistered analysis. There, evidence was inconclusive regarding the difference between the visual rotation and the other conditions. Evidence suggested no difference between the mental and visual rotation with stop (see Figure 4B).

To account for the effects of luminance changes on the mean pupil size, we performed additional exploratory analyses for the maximum pupil size corrected by the baseline. The preferred model includes both main effects of angular disparity and condition but no interaction [all log_10_(BFs) ≥ 2.992, RR = [< 0.01, >1]]. Pairwise comparisons revealed decisive evidence for a difference between the mental and both visual rotation conditions [log_10_(BF)_mental − visual_ = 7.908, log_10_(BF)_mental − visualStop_ = 18.121, RRs = [< 0.01, >1]]. Evidence regarding the difference between the visual conditions remained inconclusive [log_10_(BF) = 0.264, RR = [0.31,>1]; see Figure 4C].

#### 3.2.3 Hypothesis S2: subjective physical effort

The results for physical effort remain inconclusive [BF = −0.291, RR = [0.15, 0.63]; see Figure 4A].

#### 3.2.4 Hypothesis S3: reaction time

For reaction time, the full model with main and interaction effects, including angular disparity and condition as independent variables, is preferred with decisive evidence [all log_10_(BFs) ≥ 2.219, RR = [< 0.01, >1]]. The main effects of condition [log_10_(BF) > 100, RR = [< 0.01, >1]] and angular disparity [log_10_(BF) > 100, RR = [< 0.01, >1]] were also preferred with decisive evidence (see Figure 2B).

Pair-wise comparisons indicate strong to decisive evidence for main effect differences between all three conditions [log_10_(BF)_mental − visual_ > 100, log_10_(BF)_mental − visualStop_ > 100, log_10_(BF)_visual − visualStop_ = 1.110, all RRs = [< 0.01, >1]]. The interaction differed strongly/decisively between the mental condition and both visual conditions [log_10_(BF)_mental − visual_ = 2.936, log_10_(BF)_mental − visualStop_ = 1.123, RRs = [< 0.01, >1]], but we found strong evidence against a difference between the two visual conditions [log_10_(BF)_visual − visualStop_ = −1.572, RR = [< 0.01, >1]].

### 3.3 Exploratory analyses

#### 3.3.1 Hypothesis E1: gaze data

**Figure 5:**
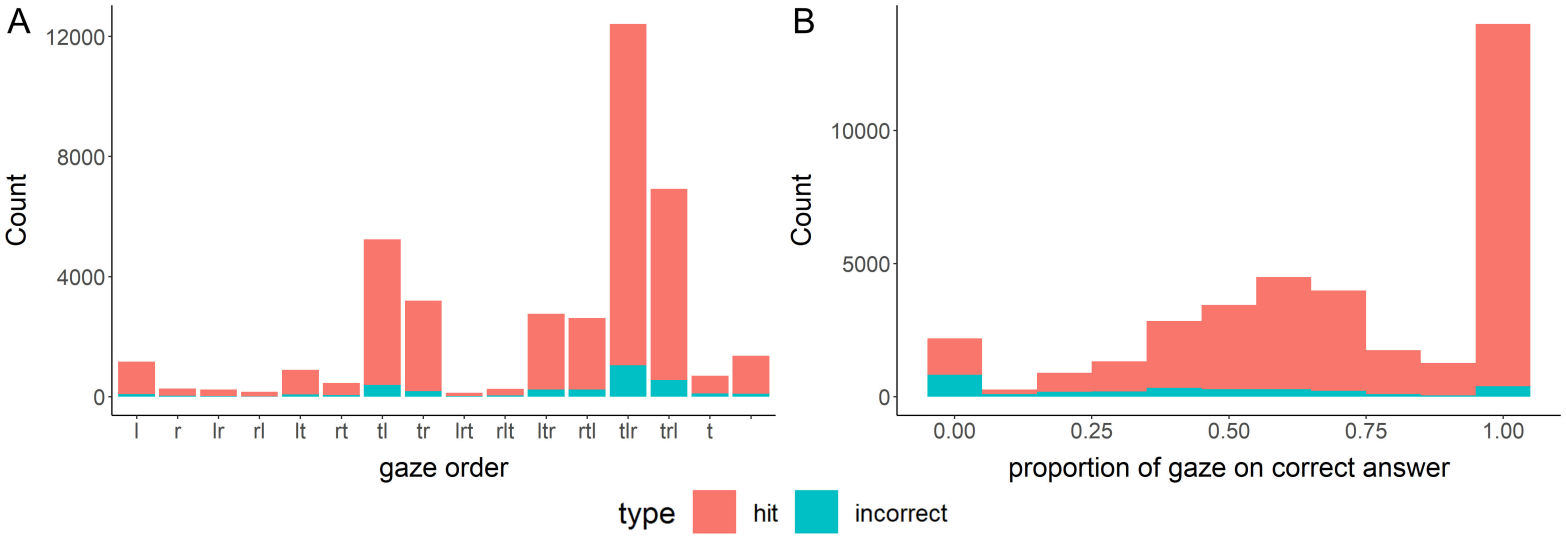
Gaze order of first focus on each figure during rotation trials **(A**, Left**)** and proportion of focus on correct alternative in the second half of trials **(B**, Right**)**.

We preregistered to explore the gaze data. First, we converted all gaze focus points, excluding blinks, into the order that the three figures were viewed by ordering the first focus on each. These data show that for 67.1% of all trials (66.9% of correct trials; by conditions: mental: 69.0, 68.6%, visual: 66.4, 66.3%, and visual with stop: 65.9, 66.0%), all three figures were viewed. As explained, this includes trials in which no focus on any alternative or the target was measured. The behavioral data implies that at least one alternative and the target were observed in most of these trials, suggesting that the actual percentage should be even higher. Regarding the order in which the alternatives were looked at, the left alternative was viewed before the right alternative in 62.2% of the trials (62.1% of correct trials; by conditions: mental: 61.9, 61.9%, visual: 62.4, 62.2%, and visual with stop: 62.2, 62.2%; see Figure 5A). When looking only at the second half of focus points in trials in which at least one alternative was focused, the gaze data indicate that the correct alternative was focused more in 71.5% of trials (21.8% focus on wrong alternative, 6.7% equal focus; by conditions: mental: 72.1, 22.3, 5.5%, visual: 71.6, 21.3, 7.1%, and visual with stop: 70.8, 21.9, 7.3%). This was larger for correct answers (74.5%) than for incorrect answers (37.2%; by conditions: mental: 76.9, 32.1%, visual: 73.8, 41.8%, and visual with stop: 73.1, 39.6%; see Figure 5B).

#### 3.3.2 Sex differences

**Figure 6:**
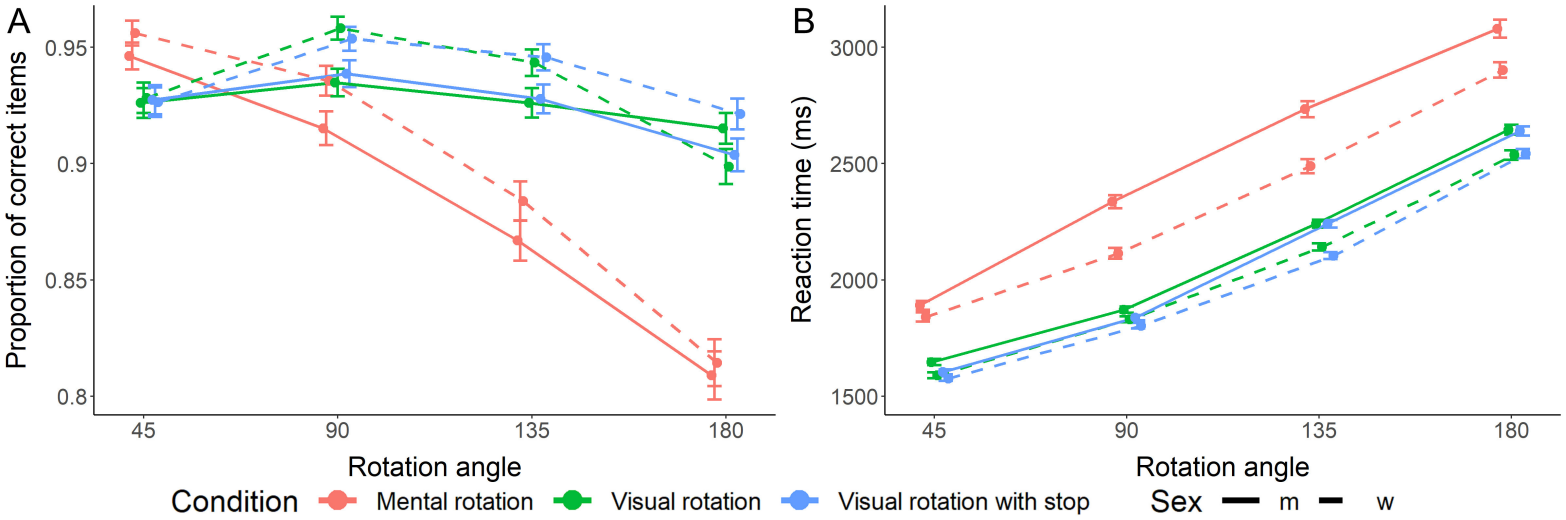
Accuracy **(A**, Left**)** and reaction time **(B**, Right**)** of rotation trials separated for men (m) and women (w). Error bars represent the standard error.

Regarding sex differences in performance, women performed better descriptively, but all relative effect sizes were negligible to small. On average, they were faster in all conditions for all angles, and they answered more trials correctly in all conditions. In the mental rotation condition, women were faster by about 170 ms (Cohen's *d* = 0.15), and accuracy was 1.3% higher (Cohen's *d* = 0.05). In the visual rotation condition, women were faster by about 80 ms (Cohen's *d* = 0.12), and accuracy was 0.7% higher (Cohen's *d* = 0.03). In the visual rotation with stop condition, women were faster by about 72 ms (Cohen's *d* = 0.12), and accuracy was 1.2% higher (Cohen's *d* = 0.05). Although sex differences varied by angular disparity, there was no monotonic relationship in any condition for neither reaction time nor accuracy (see Figure 6).

## 4 Discussion

We compared two visual rotation tests with a mental rotation test. The parameters of postural sway during the trials show no difference between conditions, suggesting similar processes. Namely, stimuli are rotated in the mind during mental rotation trials, similar to physical rotations of fixed speed. Moreover, this imagined visualization of rotation can serve as an explanation of postural sway during mental rotation.

These results are likely not due to differences in reaction time and cognitive effort between conditions. All three conditions produced different reaction times, and the slopes differed between the mental and visual conditions. Cognitive effort only differed between the mental and visual conditions.

### 4.1 Similarity of mental and visual rotation

We reproduced the patterns of postural sway during mental rotation found by Hofmann et al. (2023) and Hofmann and Jansen (2023). The mean amplitude and maximal ranges in both directions increased monotonically with angular disparity, but sway velocity stayed constant. The same effects were found in both visual rotation conditions, and the three conditions differed neither in the slope with angular disparity nor overall. The interpretation of these results is straightforward: This further supports that the same mechanism occurs in visual and mental rotation and that stimuli are indeed visualized to rotate in the mind during mental rotation trials.

Because the visual system is known to influence postural sway, Hofmann et al. interpreted the slope during mental rotation as a possible indicator that the postural sway follows the hypothesized mental rotation process. Stimuli are rotated at a constant speed but for increasing ranges. Our results further support this interpretation. However, we cannot link sway velocity to the speed of rotation because we did not vary the speed of visual rotation. A potential counterargument is that the rotation speed differed between mental and visual rotation, but the sway velocity did not. It must be noted, however, that the values obtained for sway velocity are dependent of the measurement frequency. The values measured here (and in other studies) could be superimposed by other high-frequency movements that do not allow rotation speed detection at lower frequencies.

Cognitive effort and reaction time influence measurements of postural stability (Hofmann et al., 2023). It is, therefore, possible that differences in postural sway due to reaction time and cognitive effort equalized actual differences between visual and mental rotation. For reaction time, this is implausible because both visual conditions also differed in reaction time. If reaction time influenced the results of postural sway, there should be a difference between the visual conditions. This cannot be excluded for cognitive effort because the visual conditions did not differ. However, it is still relatively unlikely that the difference in cognitive effort precisely negated differences in postural sway.

Regarding the difference between conditions, it can be argued that participants mentally rotated in all conditions. This is indicated by reaction times in the visual conditions, which are faster than when visual congruence was achieved. The visual rotation could have simply initiated or facilitated mental rotation. However, two arguments suggest that this does not impact the interpretation of postural stability. First, the faster reaction times in the visual rotation with stop condition indicates that the stopping was actually used in some trials. If all trials were solved by mental rotation, the two visual conditions should be indistinguishable in outcome measures. Second and more importantly, if they performed mental rotation during the visual rotation conditions, their postural stability was still unaffected by the visual rotation. As visual movement should influence postural sway, the likely explanation is that they were already visualizing this movement in their mind and were thus not additionally impacted.

A different argument can be made that participants were primed to visualize rotation in the mental rotation condition by the visual conditions as early as in the practice blocks. However, Hofmann and colleagues (Hofmann et al., 2023; Hofmann and Jansen, 2023) found the same pattern of results without priming, although we did not statistically compare their results and ours. Moreover, for practical purposes of testing actual mental rotation ability, such a priming might be useful to differentiate between mental rotation and other strategies. It might also be useful for training because it can be an efficient strategy if the whole shapes are rotated (Hegarty, 2018) and has been shown to improve performance (Jost and Jansen, 2022).

### 4.2 Vision as the link between movement and visuospatial abilities

Jost and Jansen (2022) already suspected that visualization is the key link between rotation movements and visuospatial abilities. However, movement is almost always linked to visual feedback on the performed action, either by observing a manipulated object or by the movement to a new location and, thus, perspective. This has not been separated in adult studies. For infants, there is also a link between movement experience and mental rotation performance (Schwarzer et al., 2013, 2022). Different forms of movement and visual feedback have been investigated. Only movements that produce rotational visual feedback improve mental rotation performance compared with rotational movements that produce non-rotational feedback (Gerhard-Samunda et al., 2021) and with non-rotational movements (Kelch et al., 2021). These support the idea that vision and not movement itself is the link to visuospatial abilities, but further studies are necessary.

In the other direction, vision and visualization also affect movement. This is known for postural stability (Lee and Lishman, 1977) but also more generally for observational learning (Han et al., 2022), motor imagery, and visualization training (Behrendt et al., 2021; Ladda et al., 2021). It is generally assumed that only cognitive training related to a movement improves motor performance, and wide transfer may not occur (e.g., Diamond and Ling, 2016). Motor learning also profits more from physically performing the actions instead of pure visual learning. Adams et al. (2014) showed a larger performance increase in a manual rotation task after manual training compared with mental rotation training. They conclude that the motor actions may require additional resources not included in purely cognitive training.

Overall, vision and visualization seem to be important links between movement and cognition. Cognitive and motor tasks may share resources, and training effects could transfer whenever the visualization of the cognitive task and the visual feedback of the motor task overlap. However, motor tasks may require additional components not included in cognitive tasks. Our results further support this account, at least for mental rotation.

### 4.3 Trial design, gaze data, and sex differences

The interpretation of the gaze data regarding the trial design is straightforward. They indicate that all three figures are viewed and used to solve the trials. Moreover, the trials are solved by comparing similar figures rather than by excluding them by finding differences.

Overall, all three figures were measurably observed in most trials across all conditions. The actual number is likely even higher because trials were solved correctly almost equally often when no gaze points on the target or on either alternative were measured. As the trials cannot be solved without knowledge of these figures, this suggests that not all observations of the figures were measured. Another indication that all figures were viewed is that participants focused more on the correct alternative during the second half of the correctly solved trials. This suggests that trials are solved by comparison rather than exclusion and in turn that the addition of the second alternative is used to solve the task. For incorrectly solved trials, this effect was reversed, albeit to a lower extent. A likely explanation for the lower effect can be guessing. Trials should be guessed correctly and incorrectly equally often, but there are more correctly solved trials than incorrectly solved trials, so the impact of guessing is larger for incorrectly solved trials. The larger focus on the chosen alternative, even for incorrectly solved trials, could suggest that not all incorrectly solved trials are due to guessing. Still, some are solved incorrectly due to wrong comparisons of figures. However, another possibility is that trials are guessed more often when the incorrect alternative is focused.

Jost and Jansen (2020) also already suspected that alternatives are viewed from left to right according to the reading direction of the participants. Our data show that the left alternative is viewed before the right alternative more often than vice versa. However, this was the case in less than two-thirds of trials. This suggests that there could be a slight impact, but further factors must be at play here. Such effects could be investigated by testing participants with other reading directions or by changing the layout of alternatives (e.g., a vertical layout of alternatives, as already mentioned by Jost and Jansen, 2020).

The gaze data also highlights problems with suspected strategy identification in chronometric tests. As all three figures are viewed in most trials, the data of the full trial cannot only be linked to the rotation strategy, but more stages must be involved. The fact that the incorrect alternative was also focused in many correctly solved trials even toward the end suggests that the last cognitive phase cannot always be a comparison phase. There is likely a mixture of phases, as Xue et al. (2017) already suggested, and/or a mixture of strategies including multiple rotation and comparison strategies (Hegarty, 2018) or also negative comparisons of mirrored figures, although this comparison is suspected to be more difficult (Paschke et al., 2012). A combination of multiple measurements, including postural sway but also, for example, fMRI, could help shed further light on this phase structure during trials. Another option is the direct measurement of phases in manual (Adams et al., 2014) or visual rotation trials. Regarding sex differences, our results align with the common observation that sex differences are non-existent or much smaller in chronometric tests, as well as in the case of three figures (Jost and Jansen, 2023; Peters and Battista, 2008). As the variance between sexes is similar across conditions, these observations also support the notion that sex differences do not occur through the mental rotation process but are caused by other aspects of the trial design unique to psychometric tests.

### 4.4 Pupil diameter

We hypothesized that pupil diameter would increase with angular disparity. The results did not support this. However, pupil diameter needs more discussion because the baseline pupil sizes were larger than the average pupil sizes throughout the trials.

We recorded eye data at a comparatively low frequency of ~13 Hz. Studies investigating pupil diameter during mental rotation specifically have used frequencies of 40–250 Hz (Bauer et al., 2021, 2022; Bochynska et al., 2021; Campbell et al., 2018). This could increase the data's variance but should average over the size of our collected sample (overall almost 1 million measurements during ~40,000 trials). Bauer et al. (2022) also observed a decline in pupil sizes at the beginning of trials, possibly due to carry-over effects due to the short break between trials (1,000 and 500 ms). At a slightly larger inter-trial interval (at least 250 ms fixation cross and 1,000 ms additional break), the data of Bochynska et al. suggest that carry-over effects should be negligible after ~1,000 ms. Nevertheless, all their data suggest that the effect of cognitive effort on pupil size should exceed any carry-over effects.

A key difference in our study is that we used brightly colored figures on a black background. Because the fixation cross during the inter-trial interval was not matched in luminance, the pupillary light reflex should have led to a decrease in pupil sizes. The onset of the pupillary light reflex is after about 200 ms and the peak response is after 500–1,000 ms (Beatty and Lucero-Wagoner, 2000; Mathôt et al., 2013) so it should have affected the mean of the trials but not the baseline measurements at the start of trials or to a much smaller extent. Because the trials differ in time, the average of longer trials should have been affected more. However, we can thus not distinguish between decreases in longer trials due to pupillary light reflex and suspected increases in longer trials due to cognitive effort. The average pupil sizes can thus not answer whether pupil diameter changes with angular disparity in mental (or visual) rotation. Note that such arguments are often not brought up when the results point in the expected direction. Even a simple control of luminance may not be sufficient as the pupillary light response is affected by attention (Mathôt et al., 2013, 2015b).

For this reason, we conducted additional exploratory analyses regarding the maximum pupil diameter within trials. The maximal pupil diameter showed the expected increase with angular disparity and the difference between the mental and visual conditions also matches the evidence for subjective effort. However, we did not find evidence for differences in slope between conditions. A possible explanation is that participants performed mental rotation in all conditions, as explained before. The increase of cognitive effort with angular disparity is in line with the studies of Bauer et al. (2021); Bauer et al. (2022) and Campbell et al. (2018) but contradicts the results of Bochynska et al. (2021). The studies of Bauer et al. also show the same pattern for maximal and mean pupil size. Similarly, Bochynska et al. obtained the same results for average pupil size and time-window analyses, and the descriptive data suggests that maximal pupil size also follows the same pattern. Our study is the only one with largely varying results, and although the luminance of stimuli may be the reason, further research is necessary. We originally planned to use mean pupil diameter because we suspected it to reflect better the overall effort invested in trials, and the maximal pupil diameter also has limitations. It is susceptible to natural fluctuations in pupil size, which occur in the time frame of 1–2 s, the same order as our trial durations (Mathôt et al., 2015a), and noise in measurements for technical reasons. Both factors should lead to increased values for measurements over longer time periods.

We need to note that some of the limitations regarding pupil size measurements and gaze data stem from the fact that we had to use proprietary and commercial software for our eye tracker. Because the hardware is no longer supported by the retailer, we had to use outdated versions of Python and OpenSesame, resulting in lower frame rates. Next to the general advantages of free open-source software, this is a specific example of an impact on data quality. Wherever possible, we encourage other researchers to use free open-source software and hardware that supports open protocols, which are also available for eye trackers (Dalmaijer et al., 2014).

### 4.5 Limitations

Hofmann et al. have noted the general limitations of force plate measurements in bipedal stance and during short-duration tasks (Hofmann et al., 2023; Hofmann and Jansen, 2023). They imply that the measurements may be influenced by factors other than postural stability and balance. Better measurements of postural stability may be obtained over longer time periods, and balance could be better measured through actually demanding tasks. However, we investigated the specific pattern and compared this between conditions. Although these may also be caused by other factors, our results strongly suggest that the same underlying mechanisms are at work.

As explained, a further limitation is that cognitive effort did not differ between visual conditions. In general, the two visual conditions were much more similar than the mental rotation condition. Thus, we cannot entirely exclude that the differences between the visual and mental condition-influenced results. However, it is unlikely that these possible other differences resulted in the exact similarity in the force plate measurements.

A question regarding the transferability of the results could arise because more than half of the participants had previous experience with mental rotation. Prior experience is often not reported for studies, but performance typically increases with experience (Jost and Jansen, 2020), which could reduce effect sizes. However, it is generally assumed that the underlying mechanisms should stay the same. Otherwise, all research focusing on naïve participants would have little application in praxis.

The low frequency of pupil size and gaze measurements also limited them. We also identified luminosity as a possible explanation for conflicting results regarding average and maximal pupil sizes, but other effects could have further interfered.

## 5 Conclusion

In conclusion, our results align with the assumption that the visualization of rotation is the reason for postural sway during mental rotation. The similarity between mental and visual rotation conditions integrates with existing evidence that visualization and visual feedback are the link between mental and manual rotation, which could transfer more generally to movement and cognition. In addition, our results demonstrate the advantages of the three-figure trial design. Measurements of pupil size indicate conflicting results regarding the effects of angular disparity, which is also apparent in the literature. Further research and better control of other effects are necessary to draw conclusive results.

## Data Availability

The datasets presented in this study can be found in online repositories. The names of the repository/repositories and accession number(s) can be found at: https://osf.io/6fj7v.

## Footnotes

^1^Note that these units may be partly arbitrary (Mathôt et al., 2018).

^2^These two participants performed below chance level in two and three conditions independent of whether outlier trials were excluded before.

^3^We preregistered to also exclude wrong answers from gaze analyses but we have split the gaze analyses for correct and incorrect answers.
