# Supplementary material for: The effect of internal and external visualization of rotation on postural stability
Source: Front Cognit. 2024 Oct 30;3:1356441. doi: 10.3389/fcogn.2024.1356441 (PMC13281227; doi:10.3389/fcogn.2024.1356441)
Supplement: Supplementary file 1 [file Data_Sheet_1.docx]

Supplementary Material

# Baseline Pupil Sizes

We planned to exclude unrealistically small baseline pupil sizes based upon visual inspection of a histogram. The histogram is shown in supplementary figure 1.

**
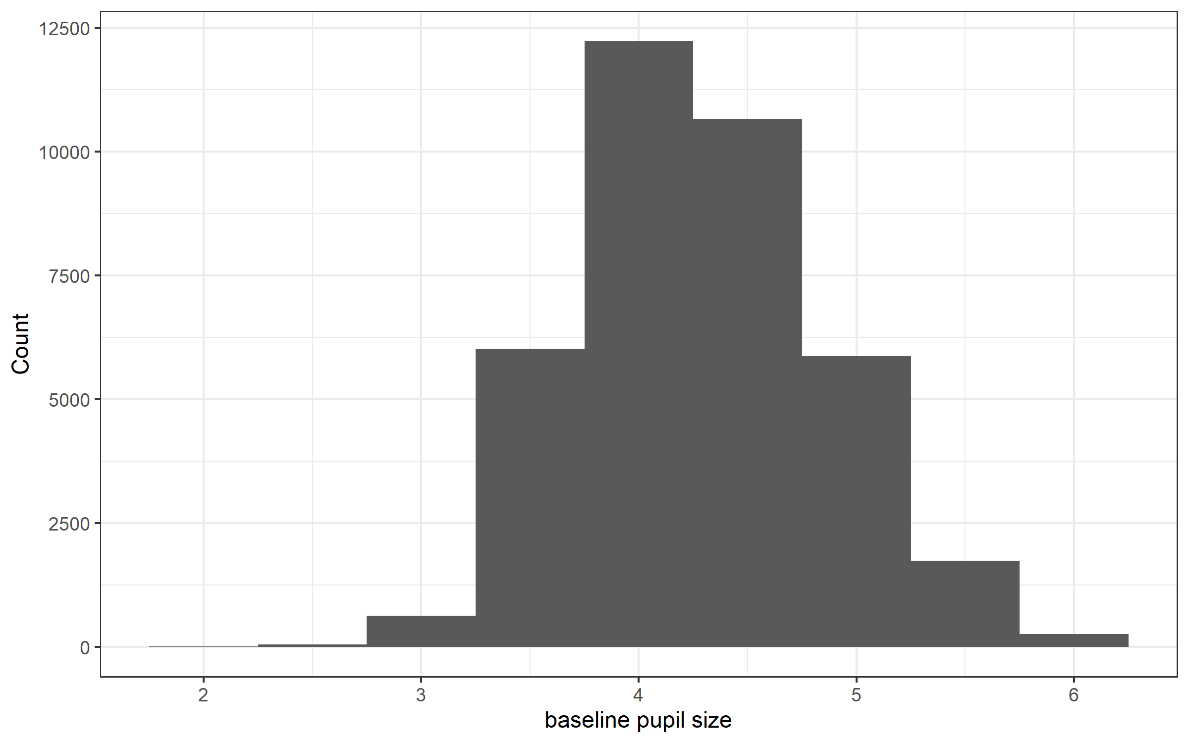
**

**Supplementary Figure 1.** Histogram of baseline pupil sizes.

# Results According to Preregistered Analysis

This section contains the results using ANOVA instead of linear models and t-tests for pairwise comparisons.

## Mean Amplitude

Analysis of mean amplitude preferred the model containing only the main effect of angular disparity (all log_10_(BFs) ≥ 3.321). The model containing both main effects was also preferred over the interaction (log_10_(BF) = 5.566). This implies decisive evidence against a condition difference both as a main effect and as an interaction with angular disparity.

## Sway Velocity

Bayes factors strongly suggest that sway velocity does not depend on angular disparity or condition (all log_10_(BFs) ≤ -1.412). The model containing both main effects was also preferred decisively over the interaction (log_10_(BF) = 6.222).

## Maximum Frontal and Sagittal Range

Like mean amplitude, the maximum frontal range is decisively implied to depend only on angular disparity but not on condition (all log_10_(BFs) ≥ 2.304) as is the maximum sagittal range (all log_10_(BFs) ≥ 3.260). In both cases, the model containing both main effects was also preferred decisively over the interaction (log_10_(BF) = 4.711 and 4.999).

## Subjective Cognitive and Physical Effort

Decisive evidence for cognitive effort to differ between conditions results from our analysis (log_10_(BF) = 86.196). Pair-wise comparisons indicate decisive evidence for differences between the mental rotation condition and both the visual condition (log_10_(BF) = 24.953) and the visual-with-stop condition (log_10_(BF) = 21.409) but substantial evidence against a difference between the two visual rotation conditions (log_10_(BF) = -0.897). Results for physical effort remain inconclusive (BF = -0.290).

## Reaction Time

For reaction time, the full model with main and interaction effects including angular disparity and condition as independent variables is preferred with decisive evidence (all log_10_(BFs) ≥ 7.594). Main effects of condition (log_10_(BF) > 100) and angular disparity (log_10_(BF) > 100) were also preferred with decisive evidence.

Pair-wise comparisons indicate strong to decisive evidence for main effect differences between all three conditions (log_10_(BF)_mental-visual_ = 22.593, log_10_(BF)_mental-visualStop_ = 22.163, log_10_(BF)_visual-visualStop_ = 0.528). We did not perform analyses whether the differences change for different angles because these rely on the slopes, which in turn require the numerical structure of angles.

## Pupil Size

The substantially preferred model for baseline-corrected pupil size includes both main effects of angular disparity and condition but no interaction (all log_10_(BFs) ≥ 0.983). Pairwise comparisons revealed inconclusive evidence for a difference between the mental and visual rotation conditions (log_10_(BF) = -0.008). Evidence suggests no difference between the mental rotation and the visual rotation with a stop (log_10_(BF) = -0.877) while evidence for the difference between the two visual rotation conditions remains inconclusive (log_10_(BF) = -0.055).
